# Whole-genome sequence data uncover loss of genetic diversity due to selection

**DOI:** 10.1186/s12711-016-0210-4

**Published:** 2016-04-14

**Authors:** Sonia E. Eynard, Jack J. Windig, Sipke J. Hiemstra, Mario P. L. Calus

**Affiliations:** Animal Breeding and Genomics Centre, Wageningen UR Livestock Research, P.O. Box 338, 6700 AH Wageningen, The Netherlands; GABI, INRA, AgroParisTech, Université Paris-Saclay, 78350 Jouy-en-Josas, France; Centre for Genetic Resources, the Netherlands, Wageningen UR, P.O. Box 338, 3700 AH Wageningen, The Netherlands

## Abstract

**Background:**

Whole-genome sequence (WGS) data give access to more complete structural genetic information of individuals, including rare variants, not fully covered by single nucleotide polymorphism chips. We used WGS to investigate the amount of genetic diversity remaining after selection using optimal contribution (OC), considering different methods to estimate the relationships used in OC. OC was applied to minimise average relatedness of the selection candidates and thus miminise the loss of genetic diversity in a conservation strategy, e.g. for establishment of gene bank collections. Furthermore, OC was used to maximise average genetic merit of the selection candidates at a given level of relatedness, similar to a genetic improvement strategy. In this study, we used data from 277 bulls from the 1000 bull genomes project. We measured genetic diversity as the number of variants still segregating after selection using WGS data, and compared strategies that targeted conservation of rare (minor allele frequency <5 %) versus common variants.

**Results:**

When OC without restriction on the number of selected individuals was applied, loss of variants was minimal and most individuals were selected, which is often unfeasible in practice. When 20 individuals were selected, the number of segregating rare variants was reduced by 29 % for the conservation strategy, and by 34 % for the genetic improvement strategy. The overall number of segregating variants was reduced by 30 % when OC was restricted to selecting five individuals, for both conservation and genetic improvement strategies. For common variants, this loss was about 15 %, while it was much higher, 72 %, for rare variants. Fewer rare variants were conserved with the genetic improvement strategy compared to the conservation strategy.

**Conclusions:**

The use of WGS for genetic diversity quantification revealed that selection results in considerable losses of genetic diversity for rare variants. Using WGS instead of SNP chip data to estimate relationships slightly reduced the loss of rare variants, while using 50 K SNP chip data was sufficient to conserve common variants. The loss of rare variants could be mitigated by a few percent (up to 8 %) depending on which method is chosen to estimate relationships from WGS data.

**Electronic supplementary material:**

The online version of this article (doi:10.1186/s12711-016-0210-4) contains supplementary material, which is available to authorized users.

## Background

The increased availability of whole-genome sequence (WGS) data allows access to more complete structural genetic information on individuals than that obtained with commonly used single nucleotide polymorphism (SNP) chips. Most SNP chips target SNPs that have approximately uniformly distributed allele frequencies [1]. In contrast, WGS data have a U-shaped distribution of allelic frequencies, with higher frequencies for rare compared to common variants [1]. Consequently, WGS data enable the estimation of relationships between individuals based on both common and rare variants, and also a more accurate estimation of the genetic diversity that is lost due to selection, across the whole range of allele frequencies. Reinforced efforts for maintaining genetic variation at rare variants are necessary because these are more likely to be lost through time, either through natural processes (i.e. drift and natural selection) or human actions (i.e. artificial selection) [2]. Rare variants can be rare due to several reasons: (1) they are linked to genetic disorders and have been (almost) purged from the population, (2) they have drifted from founder individuals and become population-specific, or (3) they are recent mutations. Rare variants can be neutral, beneficial or detrimental and be involved in complex genetic mechanisms that are so far unidentified. Importantly, rare variants may represent a source of variation that is to date not known and may be of some benefit in future breeding. Conservation of rare variants has received little attention due to the inaccessibility of most of them in common SNP chips. Because WGS data can capture both common and rare variants, its use opens new possibilities for programs on conservation of genetic diversity [3–5], in particular at rare variants that may represent one of the major focuses of management of genetic diversity in livestock species, for both long- and short-term perspectives [6].

Conservation of livestock species aims at maximising genetic diversity on the long-term. Genetic material is conserved, for example in gene bank collections, in order to allow future use or recovery of genetic variation. However, breeding programs focus mainly on genetic improvement in the next generation. Optimum contribution (OC) selection strategies have been designed to simultaneously target genetic improvement and conservation of genetic diversity. In terms of genetic diversity conservation, OC aims at minimising or restricting average relatedness of the potential parents in order to minimise the rate of inbreeding and maximise genetic diversity in the long-term [7, 8]. Previous studies [9, 10] investigated the impact of using genomic information from SNP chip data instead of pedigree information for OC and showed that adding genomic information resulted in a slightly increased genetic diversity. This improvement was more important when only a few individuals were selected from large populations [9], and when pedigree information was incomplete [11]. Simulations showed that using SNP chip data in OC selection could increase genetic gain considerably at comparable inbreeding rates [12] and that up-weighting rare alleles increased long-term genetic gain [13]. On the one hand, rare variants are expected to be more easily lost due to selection but, on the other hand, this loss may be restricted by using OC in combination with relationships derived from WGS information. Using a method based on estimated relationships that account for allele frequencies may mitigate this loss furthermore and better conserve such rare variants.

Our objective was to investigate the amount of genetic diversity conserved across the whole genome, including common and rare variants, by using OC within the context of conservation of genetic diversity and genetic improvement. Genetic diversity was measured as the number of genetic variants that still segregate in a population after selection. Relationships were estimated with different methods, using pedigree, SNP chip, or WGS data.

## Methods

### Animals

This study was performed on data from 277 Holstein bulls from Run 4 of the 1000 bulls genome project. These 277 individuals originated from Europe, North-America, Australia and New-Zealand (based on their Interbull ID) and were born between 1965 and 2010. Their full pedigree contained 12,949 individuals of which 4535 were sires and 8414 were dams, and was recorded from the 1900s onward. Base individuals in the pedigree, i.e. 3093 individuals with both parents unknown, had birth years ranging from 1883 to 2002. The average date of birth of the base individuals was 1931, while it was 1948 for the non-base individuals.

Within the group of 277 sequenced bulls, we observed 106 parent-offspring relationships, three full-sib pairs and 200 half-sib pairs. All individuals were related to some extent. Generation equivalents were computed as the sum over all ancestors of $( {\frac{1}{2}} )^{n}$, where $n$ is the number of generations between the individual and its ancestors [14], and ranged from 2.95 to 14.16 with an average of 9.91. The number of generations with complete pedigree (both sire and dam included) ranged from 1 to 8 with an average of 2.80 full generations. The pedigree completeness index ($PCI$) was computed using the ENDOG software [15] following the definition of MacCluer et al. [16]. $PCI = \frac{{2C_{sire} C_{dam} }}{{C_{sire} + C_{dam} }}$, where $C_{sire }$ and $C_{dam}$ are the paternal and maternal contribution index calculated as the proportion of ancestors $a_{i}$ known in generation $i$ divided by the number of generations known in the pedigree, as follows: $C = \frac{1}{d}\sum\nolimits_{i = 1}^{d} {a_{i} }$. The average $PCI$ was equal to 0.10 over 37 partial generations with a maximum of 0.72 for the last generation.

Required estimated breeding values (EBV) were defined as the NVI, which is the Dutch Flemish total merit index estimated by the genetic evaluation of sires for bull ranking in the Netherlands and Flanders [17]. This index combines several traits that are included in the breeding goal such as, milk production, longevity, health, fertility, and conformation. EBV from the genetic evaluation of April 2015 were available for 268 individuals of the sequenced bulls.

### Sequences

Whole-genome sequence data of the 277 bulls contained a total of 35,726,017 variants across the 29 autosomes, of which 20,177,956 segregated in this set of animals. WGS were obtained using sequencing outputs from Illumina HiSeq Systems (Illumina Inc., San Diego, CA) that were edited in five steps: sequence alignment, variant calling, phasing, quality controls and imputation. Of the called variants, 94.52 % were SNPs and 5.48 % were insertion-deletions. The overall sequence coverage per individual ranged from 3 to 38, with an average of 12. SNP-type variants that are included in the Illumina BovineSNP50 BeadChip v2 (Illumina Inc., San Diego, CA) were extracted to be used as 50 K SNP chip. This SNP subset contained 48,652 SNPs of which 46,050 were segregating in the population of 277 bulls.

### Data editing

For both the 50 K SNP chip and WGS data, we used an F-exact test of departure from Hardy–Weinberg equilibrium to estimate P-values for each of the segregating variants. In the case of low allele frequencies, i.e. when only a small number of individuals are allocated to one of the genotype classes, the F-exact test has been shown to be the most suitable method [18] to assess departure from Hardy–Weinberg equilibrium. In total 313,241 and 68 variants that departed from Hardy–Weinberg equilibrium, after Bonferroni correction for multiple testing [19], were removed from the WGS and 50 K SNP chip data respectively (P-values <10^−10^ for WGS and <10^−6^ for 50 K SNP chip data). Moreover, variants that had a minor allele frequency (MAF) lower than 1 % were also excluded since they are more likely to represent genotyping errors rather than true variants. This threshold was equivalent to removing variants for which the rare allele was present less than 6 times in our data set. This step removed 4,000,558 variants from the WGS and 1615 from the SNP chip data. After all editing, a set of 15,864,157 variants for WGS data and 44,367 variants for the 50 K SNP chip remained for our analyses.

### Optimal contribution

Selection based on optimal contribution (OC) was performed, using the program Gencont [7], for conservation alone (*cons*), or combined genetic improvement and conservation (*impcons*). In both selection strategies, estimated relationships between selection candidates were computed using pedigree, 50 K SNP chip or WGS data. OC jointly maximises conservation of genetic diversity and genetic gain, by optimising the contribution of the selection candidates while minimising the rate of inbreeding in the next generation $\left( {t + 1} \right)$ and in the long-term. These parameters can be defined as follows:

- The average coancestry between selected individuals, since it represents the change in inbreeding between the current and next generation, $\overline{{{\text{r}}_{t + 1} }}$:
  $$\overline{{{\text{r}}_{{{\text{t}} + 1}} }} = \frac{{{\mathbf{c}}_{\text{t}}^{ '} {\mathbf{A}}_{\text{t}} {\mathbf{c}}_{\text{t}} }}{2}$$
  or
  $$\overline{{{\text{r}}_{{{\text{t}} + 1}} }} = \frac{{{\mathbf{c}}_{\text{t}}^{ '} {\mathbf{G}}_{\text{t}} {\mathbf{c}}_{\text{t}} }}{2}$$
- The average genetic merit of the next generation, $\overline{M_{t+1}}$:
  $$\overline{M_{t + 1}} = {\mathbf{c}}_{\text{t}}^{ '} {\mathbf{EBV}}_{\text{t}} ,$$
  where ${\mathbf{c}}_{\text{t}}$ is the vector of genetic contributions of the selected individuals, ${\mathbf{A}}_{\text{t}}$ and ${\mathbf{G}}_{\text{t}}$ are the additive genetic and genomic relationship matrices, and ${\mathbf{EBV}}_{\text{t}}$ is a vector of estimated breeding values.

The algorithm behind the determination of the OC ${\mathbf{c}}_{\text{t}}$ that maximises genetic diversity and genetic gain with the aforementioned constraints is explained in more detail in [7].

In our study, there were nine individuals with missing EBV, which were marked as unavailable for selection. We optimised genetic contribution of the remaining 268 individuals by: (1) minimising the average relatedness and thereby minimising the rate of inbreeding in the long-term while genetic gain was not constrained (hereafter referred to as *cons* since it targets conservation only), or (2) maximising genetic gain and setting the rate of inbreeding $\Delta F$ to the standard value of 0.01 per generation [20] (hereafter referred to as *impcons* since it targets genetic improvement and conservation). In all cases, we estimated $\overline{M_{t + 1}}$ as the average genetic merit of the group of individuals that remained after selection.

### Estimation of relationships

The method for OC requires relationships between individuals in the current population. Therefore, additive genetic (${\mathbf{A}}$) and genomic (${\mathbf{G}}$) relationship matrices were calculated on the 277 individuals. Currently, there is no consensus on which method should be used to calculate ${\mathbf{G}}$-matrices in the context of genetic diversity [9, 10, 21]. Our aim was to select the methods to estimate relationships that had the highest potential for maintaining genetic diversity. Therefore, ${\mathbf{G}}$-matrices were calculated in four different ways, as explained below.

1. According to the first method described by VanRaden [22]:
  $$G_{jk} = \frac{{\mathop \sum \nolimits_{\text{i}} \left( {x_{ij} - 2p_{i} } \right)\left( {x_{ik} - 2p_{i} } \right)}}{{2\mathop \sum \nolimits_{\text{i}} p_{i} \left( {1 - p_{i} } \right)}}$$
2. According to the second method described by VanRaden [23]:
  $$G_{jk} = \frac{1}{N}\mathop \sum \limits_{i} \frac{{\left( {x_{ij} - 2p_{i} } \right)\left( {x_{ik} - 2p_{i} } \right)}}{{2p_{i} \left( {1 - p_{i} } \right)}}$$
  In these two formulas, $N$ is the number of variants and $G_{jk}$ is the estimated relationship between individuals $j$ and $k$ across loci. At each locus $i$, $x_{i} .$ is the individual variant genotype coded as 0, 1 or 2 and $p_{i}$ is the frequency of the allele for which the homozygous genotype is coded as 2 at locus $i$.
3. We used Yang’s method [24] as an alternative to VanRaden’s [23] second method:
  $$G_{jk} = \frac{1}{N}\mathop \sum \limits_{i} G_{ijk} = \left\{ {\begin{array}{ll} {\frac{1}{N}\mathop \sum \limits_{i} \frac{{\left( {x_{ij} - 2p_{i} } \right)\left( {x_{ik} - 2p_{i} } \right)}}{{2p_{i} \left( {1 - p_{i} } \right)}}, \quad j \ne k} \\ {1 + \frac{1}{N}\mathop \sum \limits_{i} \frac{{x_{ij}^{2} - \left( {1 + 2p_{i} } \right)x_{ij} + 2p_{i}^{2} }}{{2p_{i} \left( {1 - p_{i} } \right)}},\quad j = k} \\ \end{array} } \right.$$
  In this case off-diagonal elements are computed as in VanRaden’s second method, while diagonals are computed by considering that self-relationships are expected to be equal to 1 plus inbreeding. Both VanRaden’s second and Yang’s methods have similar properties, with the only difference being that, in Yang’s method, self-relationships are computed more precisely. Because diagonal and off-diagonal elements are computed differently non semi-positive definite matrix can be obtained with Yang’s method. All genomic matrices involved allele frequencies $p_{i}$ that were estimated based on the current population of 277 bulls.
4. Finally, genomic relationships were computed without using information on allele frequency, i.e. we used either of the first three ${\mathbf{G}}$-matrices described above with all $p_{i}$ values set to 0.5 [1]. Note that this yields equivalent results to the methods that were initially proposed by Nejati-Javaremi et al. [25] and by Eding and Meuwissen [26]. These estimated relationships, which count the number of identical alleles averaged across loci between two individuals, are equivalent except that the scales are different. Such similarity-based methods have also been applied in other studies [9, 10].

Using VanRaden’s second method instead of Yang’s method allowed us to investigate for potential issues in the calculation of OC that could be due to the non semi-positive definite matrix. The OC algorithm was entirely run with all four matrices. However, both VanRaden’s methods generally performed slightly less well than Yang’s or the similarity-based methods in terms of conservation of genetic diversity and were therefore discarded in the remaining analyses (see Additional file 1 for a comparison of all four methods).

### Measure of genetic diversity

Whether for inclusion in a gene bank or for use in breeding programs, using all individuals with non-zero contributions, weighted by these contributions, is often not feasible and the aim becomes to select a subset of all available selection candidates. Thus, OC with a restriction on the number of selected individuals, assuming that they contribute equally to the next generation is often used instead. We either used the traditional OC without restriction on the number of individuals selected, or OC with a restriction set to select 20, 10 or 5 individuals. We compared the number of variants that segregated in groups of selected individuals after performing OC selection to the total number of variants before selection [27–29]. The results were evaluated for three categories of variants: rare variants (MAF between 1 and 5 %), common variants (MAF ≥ 5 %) and all variants (MAF ≥ 1 %). A summary of the different variables and values considered in the analysis is in Table 1.

**Table 1:** Variables and values considered across the different scenarios

Variables	Values taken
Selection strategies	Conservation (*cons*), genetic improvement and conservation (*impcons*)
Rate of inbreeding	Minimised, 1 %
Estimated relationships	A, SNP_Yang, SNP_Similarity, WGS_Yang, WGS_Similarity
Restriction on number of selected individuals	No, 20, 10, 5
Variants	All, Common, Rare

In both *cons* and *impcons* strategies, the resulting average genetic merit was evaluated.  Rates of inbreeding were calculated according to the formula from Falconer and Mackay [30]:

$$\Delta F = \frac{{F_{t + 1} - F_{t} }}{{1 - F_{t} }} = \frac{{\overline{{{\mathbf{A}}_{{{\text{t}} + 1}} }} - \overline{{{\mathbf{A}}_{\text{t}} }} }}{{2 - \overline{{{\mathbf{A}}_{\text{t}} }} }}\;{\text{or}}\;\frac{{\overline{{{\mathbf{G}}_{{{\text{t}} + 1}} }} - \overline{{{\mathbf{G}}_{\text{t}} }} }}{{2 - \overline{{{\mathbf{G}}_{\text{t}} }} }}$$

$F_{t}$ and $F_{t + 1}$ are the average inbreeding coefficients in generations $t$ and $t + 1$, respectively, and were calculated as half the average relationship in the group of individuals before ($\overline{{{\mathbf{A}}_{\text{t}} }}$ and $\overline{{{\mathbf{G}}_{\text{t}} }}$) and after selection ($\overline{{{\mathbf{A}}_{\text{t + 1}} }}$ and $\overline{{{\mathbf{G}}_{{{\text{t}} + 1}} }}$). In all cases, the rates of inbreeding were calculated based on the relationship matrix used for selection and also on the four relationship matrices described above. It is important to note, that using different methods to estimate relationships can lead to different scales of the estimates [31]. As a result, the inbreeding levels calculated for the current generation that are used to compute the rate of inbreeding, are also evaluated on different scales.

Methods that account for allele frequencies such as VanRaden’s methods and Yang’s method should preferably be based on the allele frequencies in the base population. In practice, since it is complicated to obtain such information, allele frequencies calculated for the current population are often used instead. One way to standardize the scales across different types of estimated relationships is to rescale the considered genomic relationship matrices ${\mathbf{G}}$ (calculated for the current population of genotyped animals) to the scale of the pedigree relationship matrix ${\mathbf{A}}$ (calculated for the old base population at the start of the known pedigree). Transformations have been proposed for instance by Forni et al. [32] and Meuwissen et al. [33]. In our study, we initially considered the transformation from Vitezica et al. [34], which is equivalent to the transformation from Powell et al. [35], to rescale ${\mathbf{G}}$ and ${\mathbf{A}}$-matrix to an equivalent base population. Vitezica’s transformation is as follows:

$${\mathbf{G}}^{*} = \left( {1 - \frac{1}{2}\alpha } \right){\mathbf{G}} + \alpha ,$$

with

$$\alpha = \frac{1}{{n^{2} }}\left( {\sum {\mathbf{A}} - \sum {\mathbf{G}}} \right),$$

where *n* is the number of individuals and ${\mathbf{G}}^{*}$ is the ${\mathbf{G}}$-matrix corrected to match the base population.Alternatively, these transformations can be applied directly to the formula of $\Delta F$ instead of to the ${\mathbf{G}}$-matrix. Using the transformation of Vitezica et al. [34], the formula for the rate of inbreeding then becomes:

$$\begin{aligned}\Delta F^{*} &= \frac{{\overline{{{\mathbf{G}}_{{{\text{t}} + 1}}^{ *} }} - \overline{{{\mathbf{G}}_{\text{t}}^{ *} }} }}{{2 - \overline{{{\mathbf{G}}_{\text{t}}^{ *} }} }} \\ &= \frac{{\left( {\left( {1 - \frac{1}{2}\alpha } \right)\overline{{{\mathbf{G}}_{{{\text{t}} + 1}} }} + {{\alpha }} - \left( {1 - \frac{1}{2} \alpha } \right)\overline{{{\mathbf{G}}_{\text{t}} }} - \alpha } \right)}}{{\left( {2 - \left( {1 - \frac{1}{2}\alpha } \right)\overline{{{\mathbf{G}}_{\text{t}}}} - \alpha } \right)}} \\ &= \frac{{\left( {\overline{{{\mathbf{G}}_{{{\text{t}} + 1}} }} - \overline{{{\mathbf{G}}_{\text{t}}}}} \right)}}{{\left( {2 - \overline{{{\mathbf{G}}_{\text{t}}}}} \right)}} =\Delta F \\ \end{aligned}$$

In our case, using this or any other linear transformation did not affect the level of contribution whether based on average coancestry or rate of inbreeding; therefore we used the untransformed ${\mathbf{G}}$-matrices in this study.

## Results

### Genetic variation and genetic merit before selection

The estimated relationships obtained with the similarity-based method were higher and less variable than those based on pedigree and genomic data using Yang’s method (Table 2). Across the 277 bulls used in this study, the total number of variants (MAF ≥ 1 %) was equal to 15,864,157, with 11,449,016 common variants (MAF ≥ 5 %) and 4,415,141 rare variants (MAF between 1 and 5 %). Across the 268 individuals that were available for selection, the total number of variants was equal to 15,857,694 (11,448,863 common and 4,408,831 rare variants), which means that only 0.04 % of these were absent in the genome of the individuals used for the investigation. EBV for these 268 individuals ranged from −295 to 192 with an average of −61.

**Table 2:** Descriptive statistics of the estimated relationships

Data type and estimator	Minimum	Mean	Maximum	Variance
*Self-relationships (n = 277)*				
A	1.00	1.03	1.17	0.00065
SNP_Yang	0.70	0.99	1.13	0.00185
SNP_Similarity	1.03	1.30	1.39	0.00111
WGS_Yang	0.78	0.94	1.05	0.00111
WGS_Similarity	1.35	1.50	1.56	0.00069
*Relationships between individuals (n = 38,226)*				
A	0.00	0.07	0.67	0.00333
SNP_Yang	−0.12	0.00	0.65	0.00305
SNP_Similarity	0.48	0.60	1.04	0.00231
WGS_Yang	−0.08	0.00	0.58	0.00212
WGS_Similarity	0.93	1.02	1.30	0.00128

Minimum, mean, maximum and variance of the estimated relationships calculated based on pedigree, 50 K SNP chip (SNP) or whole-genome sequence (WGS) data with Yang’s method [24] or the similarity-based method

### Genetic diversity in the conservation strategy (*cons*)

**Fig. 1:**
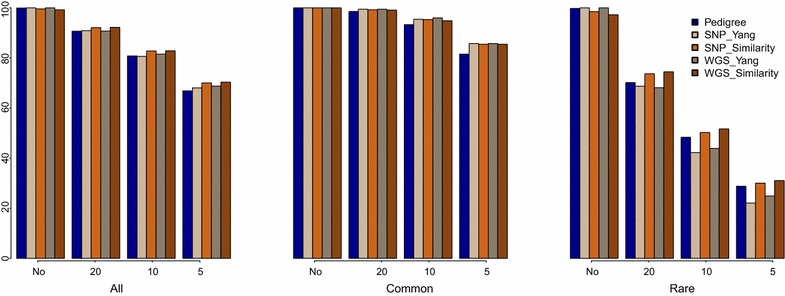
**Segregating variants after selection for conservation (cons)**. Relationships are computed based on pedigree, 50 K SNP chip (SNP) or whole-genome sequence (WGS), using either the method described by Yang et al. [24] or the similarity-based method. The first histogram is for all variants (MAF ≥ 1 %), the second is for common variants (MAF ≥ 5 %) and the last is for rare variants (MAF between 1 and 5 %). In *each histogram*, the first block corresponds to the case without constraint on the number of selected individuals, the second, third and fourth histograms correspond to the cases that constrain the number of selected individuals to 20, 10 and 5, respectively

When no restriction was put on the number of selected individuals and the estimated relationships based on pedigree information were used, a subset of 128 individuals was selected and individual contributions to the next generation ranged from 0.006 to 3.628 % (Table 3). Using estimated relationships based on either SNP chip or WGS data computed with Yang’s method ended in selecting all 268 individuals, and thus, all available variants were conserved within this population. Individual contributions to the next generation ranged from 0.172 to 0.708 %. In contrast, using similarity-based estimated relationships led to the selection of a subset of 89 individuals when they were based on SNP chip data and 71 individuals when they were based on WGS data, with contributions to the next generation ranging from 0.004 to 9.076 %. The overall percentage of segregating variants after selection ranged from 99.23 to 100 % depending on the type of data and method used to estimate relationships. The percentage of common variants segregating after selection was always 100 %. The percentage of rare variants segregating after selection ranged from 97.24 to 100 % depending on the type of data and method used to estimate relationships (Fig. 1).

**Table 3:** Individual contributions (as percentage) in each of the selection strategies without restriction on the number of selected individuals

Strategy	Data type and estimator	Number of selected individuals	Min	Mean	Max
*cons*	A	128	0.006	0.781	3.628
	SNP_Yang	268	0.276	0.373	0.708
	SNP_Similarity	89	0.004	1.124	9.076
	WGS_Yang	268	0.172	0.373	0.617
	WGS_Similarity	71	0.060	1.409	7.944
*impcons*	A	34	0.095	2.941	7.646
	SNP_Yang	84	0.015	1.191	4.180
	SNP_Similarity	39	0.012	2.564	6.604
	WGS_Yang	85	0.011	1.176	4.240
	WGS_Similarity	32	0.068	3.125	11.866

Contributions are expressed as the percentage of the offspring produced in the next generation; the mean was calculated on the individuals having a contribution >0

If restrictions were set on the number of selected individuals, the percentages of variants changed as follows: with 20, 10 and 5 selected individuals, 98.55 to 99.44, 93.29 to 96.00 and 81.54 to 85.77 % of the common variants and 68.14 to 74.44, 42.23 to 51.68 and 22.05 to 31.03 % of the rare variants segregated, respectively. Under these conditions, the relationships estimated by Yang’s method based on SNP chip data performed best to conserve common variants (from 99.44 to 85.77 % depending on the number of selected individuals), although the differences with other combinations of method and data type were small. For rare variants, similarity-based estimated relationships using WGS data performed best to maintain them in the population (from 74.44 to 31.03 % depending on the number of selected individuals) (Fig. 1).

### Genetic diversity in the genetic improvement and conservation strategy (*impcons*)

**Fig. 2:**
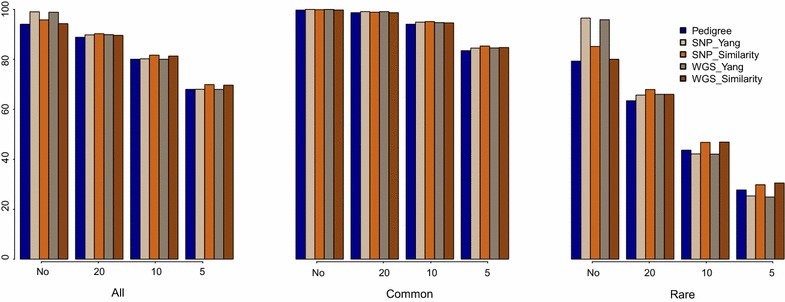
**Segregating variants after selection for genetic improvement and conservation (impcons)**. Relationships are computed based on pedigree, 50 K SNP chip (SNP) or whole-genome sequence (WGS), using either the method described by Yang et al. [24] or the similarity-based method. The first histogram is for all variants (MAF ≥ 1 %), the second for common variants (MAF ≥ 5 %) and the last for rare variants (MAF between 1 and 5 %). In *each histogram*, the first block corresponds to the case without constraint on the number of selected individuals, the second, third and fourth histograms correspond to the cases that constrain the number of selected individuals to 20, 10 and 5, respectively

When no restriction was put on the number of selected individuals, using estimated relationships based on pedigree information resulted in selecting a subset of 34 individuals (Table 3). Individual contributions to the next generation varied from 0.095 to 7.646 %. Estimated relationships based on either SNP chip or WGS data and computed with Yang’s method resulted in selecting 84 and 85 individuals, respectively, and individuals contributions to the next generation ranged from 0.011 to 4.240 %. Using similarity-based estimated relationships ended in selecting only a subset of 39 or 32 individuals using SNP chip or WGS data, with contributions to the next generation ranging from 0.012 to 11.866 %. After selection, the proportions of all segregating variants, common and rare variants ranged from 94.05 to 99.03, 99.74 to 100 and 79.29 to 96.50 % depending on the type of data and method used to estimate relationships (Fig. 2).

If restrictions were set on the number of selected individuals, the percentage of variants changed as follows: with 20, 10 and 5 selected individuals, 98.66 to 99.11, 94.07 to 95.15 and 83.51 to 85.35 % of the common variants, and 63.40 to 67.94, 42.11 to 46.93 and 24.91 to 30.50 % of the rare variants segregated after selection. In these conditions, in general, estimated relationships based on similarity and calculated from SNP chip data performed best to conserve common variants (from 98.89 to 85.35 % depending on the number of selected individuals), while similarity-based estimated relationships calculated from WGS data performed best to conserve rare variants (from 66.02 to 30.50 % depending on the number of selected individuals) (Fig. 2).

### Genetic merit and rate of inbreeding

**Fig. 3:**
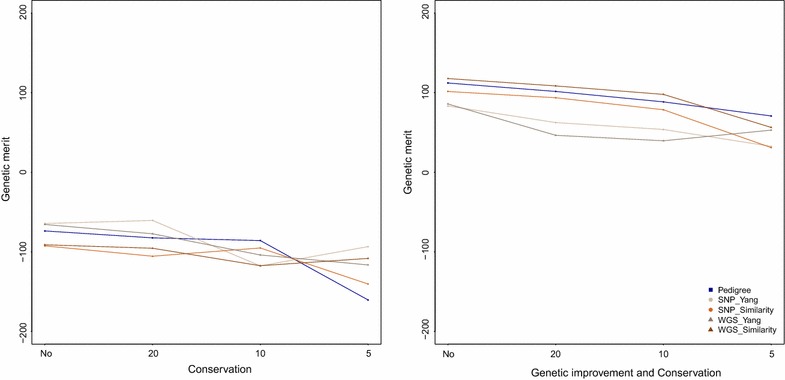
**Average genetic merit after selection for conservation (cons), and genetic improvement and conservation** ***(impcons)*** **strategies**. The *dark blue symbol* and *line *represent the full pedigree, the *beige* represent the scenario for which Yang’s estimated relationships from SNP chip data are used, the *orange* represent the scenario for which similarity-based estimated relationships from SNP chip are used, the *dark beige* represent the scenario Yang’s estimated relationships from WGS data are used, finally, the *brown* represent the scenario for which similarity-based estimated relationships from WGS data are used. The *first plot* represents the evolution of average genetic merit when the constraint on the number of selected individuals goes from none to 20, 10 and 5 in the strategy *cons*, with minimised $\Delta F$, the *second plot* represents the evolution of average genetic merit when the constraint on the number of selected individuals goes from none to 20, 10 and 5 in the strategy *impcons*, with $\Delta F$ fixed to 0.01

When the rate of inbreeding was minimised in the *cons* strategy, the average genetic merit after selection was always negative and ranged from −160.40 to −60.50 (Fig. 3). Using the relationships estimated with Yang’s method, the loss in terms of average genetic merit was smallest. For the *impcons* strategy, with a rate of inbreeding set to 1 %, average genetic merit ranged from 31.00 to 117.81 (Fig. 3). In general the genetic merit decreased as the number of selected individuals decreased. Using estimated relationships computed with the similarity-based method and WGS data resulted in the highest genetic merit.

In all cases, the rate of inbreeding increased as the number of selected individuals decreased. For the *cons* strategy, $\Delta F$ increased by 0.8 to 1.4 % (no restriction to 20 individuals selected), by 2.4 to 3.4 % (no restriction to 10 individuals selected), and by 5.8 to 8.3 % (no restriction to five individuals selected) depending on the type of data and method used. For the *impcons* strategy, $\Delta F$ increased by 0.07 to 0.95 % (no restriction to 20 individuals selected), by 0.34 to 3.00 % (no restriction to 10 individuals selected), and by 3.54 to 7.52 % (no restriction to five individuals selected) depending on the type of data and method used. In general, the rate of inbreeding was lowest or closest to our target of 1 %, when the same type of information was used both for selection and to compute the rate of inbreeding (Tables 4, 5), which agrees with the findings of Sonesson et al. [21]. In a few cases, rates of inbreeding were lowest if the same estimated relationship method (Yang’s or similarity-based) but different types of data (WGS or SNP chip) were used for calculation. Negative rates of inbreeding were observed when the level of relationships among the individuals that were selected to produce the next generation was lower than the average level of the current population. For the *impcons* strategy, the 1 % rate of inbreeding was only met when no restriction on the number of selected individuals was applied. When combining all these results together for the *cons* strategy, which minimised $\Delta F$, we observed that using similarity-based estimated relationships calculated from WGS data resulted in the lowest rates of inbreeding. In the *impcons* strategy, the rates of inbreeding were lowest when using similarity-based estimated relationships calculated from either SNP chip or WGS data.

**Table 4:** Rate of inbreeding for conservation (*cons*) strategy, based on different types of estimated relationships

Restriction	Data type and estimator	ΔF_A	ΔF_SNP_Yang	ΔF_SNP_Similarity	ΔF_WGS_Yang	ΔF_WGS_Similarity
No restriction	A	*−0.015*	0.013	−0.007	0.012	−0.013
	SNP_Yang	0.002	*0.002*	0.002	0.002	0.002
	SNP_Similarity	0.002	0.018	*−0.020*	0.017	−0.021
	WGS_Yang	0.002	0.002	0.002	*0.002*	0.003
	WGS_Similarity	−0.003	0.018	−0.013	0.019	**−** ***0.031***
20 selected	A	*−0.003*	0.038	0.004	0.035	−0.006
	SNP_Yang	0.028	*0.015*	0.012	0.016	0.009
	SNP_Similarity	0.008	0.027	*−0.011*	0.025	−0.015
	WGS_Yang	0.027	0.015	0.013	*0.015*	0.012
	WGS_Similarity	0.007	0.030	−0.002	0.030	**−** ***0.023***
10 selected	A	*0.019*	0.064	0.031	0.059	0.023
	SNP_Yang	0.048	*0.033*	0.026	0.034	0.032
	SNP_Similarity	0.034	0.047	*0.005*	0.046	−0.002
	WGS_Yang	0.048	0.034	0.030	*0.034*	0.024
	WGS_Similarity	0.032	0.053	0.011	0.052	**−** ***0.006***
5 selected	A	*0.069*	0.118	0.092	0.108	0.084
	SNP_Yang	0.107	*0.073*	0.073	*0.073*	0.070
	SNP_Similarity	0.090	0.088	*0.038*	0.087	0.034
	WGS_Yang	0.094	0.074	0.065	0.075	0.061
	WGS_Similarity	0.090	0.091	0.041	0.089	***0.029***

The lowest estimated rates of inbreeding calculated from each type of estimated relationship matrix depending on the scenario are in italics. The overall lowest value of estimated rate of inbreeding is in italic bold

**Table 5:** Rate of inbreeding for genetic improvement and conservation (*impcons*) strategy, based on different types of estimated relationships

Restriction	Data type and estimator	ΔF_A	ΔF_SNP_Yang	ΔF_SNP_Similarity	ΔF_WGS_Yang	ΔF_WGS_Similarity
No restriction	A	*0.010*	0.022	0.022	0.021	0.020
	SNP_Yang	0.011	*0.010*	0.014	*0.010*	0.011
	SNP_Similarity	0.016	0.022	*0.010*	0.021	***0.006***
	WGS_Yang	0.012	0.011	0.015	0.010	0.013
	WGS_Similarity	0.025	0.030	0.022	0.029	0.010
20 selected	A	*0.011*	0.026	0.026	0.025	0.022
	SNP_Yang	0.022	*0.019*	0.022	0.019	0.014
	SNP_Similarity	0.018	0.027	*0.011*	0.025	***0.003***
	WGS_Yang	0.023	0.020	0.022	*0.019*	0.016
	WGS_Similarity	0.022	0.028	0.019	0.027	0.011
10 selected	A	*0.028*	0.052	0.045	0.051	0.029
	SNP_Yang	0.047	*0.040*	0.047	*0.039*	0.036
	SNP_Similarity	0.039	0.045	*0.024*	0.043	0.015
	WGS_Yang	0.044	0.041	0.048	0.040	0.036
	WGS_Similarity	0.035	0.048	0.029	0.048	***0.013***
5 selected	A	*0.075*	0.107	0.085	0.102	0.069
	SNP_Yang	0.086	0.085	0.088	0.083	0.071
	SNP_Similarity	0.077	0.094	*0.057*	0.089	***0.045***
	WGS_Yang	0.088	*0.083*	0.087	*0.080*	0.072
	WGS_Similarity	0.075	0.101	0.064	0.096	0.045

The lowest estimated rates of inbreeding calculated from each type of estimated relationship matrix depending on the scenario are in italics. The overall lowest value of estimated rate of inbreeding is in italic bold

### Comparison of strategies

No major differences were observed between the *cons* and *impcons* strategies regarding loss of common variants. However, a clear decrease in the number of segregating rare variants was observed between these two strategies. On average, 11.72 % more rare variants were lost with the *impcons* strategy without restriction on the number of selected individuals than with the *cons* strategy. This loss was smaller when setting a restriction on the number of selected individuals (20, 10 and 5) because, applying such a restriction, greatly reduced the number of segregating rare variants from the beginning. Rate of inbreeding followed a similar trend for both *cons* and *impcons* strategies and increased as the restriction on the number of selected individuals became more stringent. Selecting for genetic improvement and conservation caused a slightly larger loss of genetic diversity but a major genetic gain compared to selecting for conservation only.

## Discussion

In this study, we assessed which type of data: pedigree, SNP chip or WGS, and which method should be used to reach optimal conservation of genetic diversity, measured as the number of WGS variants still segregating after selection. We were interested in two strategies that both used OC: selection for conservation only, e.g. to enrich gene bank collection (*cons*), and selection for genetic improvement while restricting loss of genetic diversity, in breeding programs (*impcons*). For both strategies, we observed a dramatic loss of genetic diversity at rare variants due to selection.

### Data

The data used in our study were either data that are currently widely used in animal breeding, i.e. pedigree or genomic data from a 50 K SNP chip, or WGS. Both types of data have some disadvantages. First, one of the major issues is the quality of the pedigree records. In fact, the more complete and deep is a pedigree, the more accurate are the estimated relationships between individuals, and thus, a more accurate OC selection can be performed [11]. To substantiate this, we compared results from three pedigree subsets that differed in depth and completeness (see Additional file 2). We observed that when most of the individuals were kept after selection, the completeness and depth of the pedigree did not have a considerable impact, but when the restriction on the number of individuals selected was more stringent (i.e. only 10 to 5 selected individuals), the most complete pedigree was best for maintaining genetic diversity conservation and especially for rare variants. This shows that when the restriction on the number of individuals to be selected becomes more stringent, accurate information on the relationships between individuals becomes increasingly important to precisely select the least related individuals.

Second, it is expected that realised relationships between individuals based on genomic data will be more accurate [36, 37] than those based on pedigree data, because genomic data cover information at the variant level. WGS data are not yet commonly used for animal breeding due to issues related to data acquisition, handling and storage. In spite of these issues, WGS data have some interesting characteristics i.e. they are not affected by ascertainment bias [38] and therefore give a lot more information on rare variants. Such rare variants are often ignored because they may lead to genotyping errors [39, 40]. In this study, quality controls were applied in the analysis to reduce the risk of using apparent segregating variants that are in fact induced by genotyping errors. We focused on comparing WGS data with more common data such as pedigree and SNP chip data in order to investigate their potential for conservation of genetic diversity.

### Different relationship estimators

**Fig. 4:**
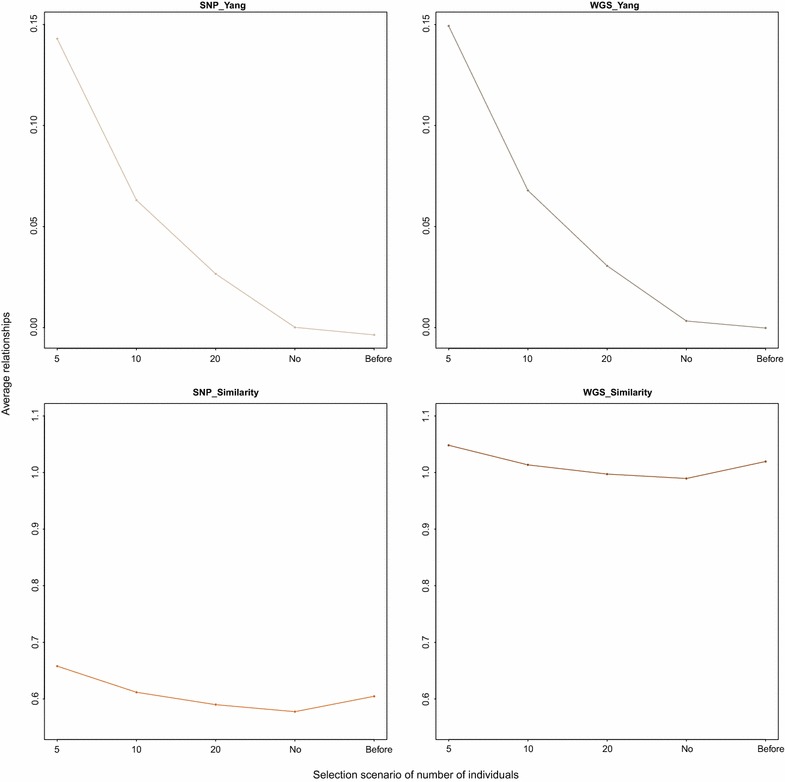
**Evolution of the average relationship of the selected group for conservation (cons) strategy**. *Each plot* represents the evolution of average relationship in the group of selected individuals in the *cons* strategy. The plots on the first row correspond to the use of Yang’s estimated relationships from SNP and WGS data, respectively and the plots on the second row to the use of similarity-based estimated relationships from SNP and WGS data, respectively

Our results, in agreement with results of de Cara et al. [10] and Engelsma et al. [9], showed that estimated relationships based on genomic data slightly outperformed those based on pedigree data for genetic diversity conservation. We expected that Yang’s method which gives higher weight to the rare variants would be the most efficient in maintaining rare variants [1], and therefore, would be more suitable for genetic diversity conservation measured on WGS data. Our results showed that Yang’s method did indeed result in a higher level of conserved genetic diversity when there was no restriction on the number of selected individuals and on rate of inbreeding levels. However, this was achieved because all available individuals were kept in the population. In contrast, the similarity-based method resulted in only a subset of individuals being kept. These differences can be explained as follows: OC minimises the average relatedness of selected individuals including self-relatedness. On the one hand, Yang’s method resulted in a low average relatedness between individuals (on average 0.00) compared to the self-relationships (on average 0.97). On the other hand, with the similarity-based method, the difference between average relatedness between individuals (on average 0.80) and self-relatedness (on average 1.40) was smaller. As a result, with Yang’s method the average relatedness of the selected individuals tends to decrease continuously when more individuals are added to the selected group, whereas with the similarity-based method, at some stage, the average relatedness reaches a minimum value and increases thereafter (Fig. 4). Hence, if there is no restriction on the number of individuals to be selected, more individuals are selected when relationships are estimated with Yang’s method than with the similarity-based method. However, if there is a restriction on the number of selected individuals, the number of conserved rare variants is larger with the similarity-based method than with Yang’s method. Due to weighing of the variants in Yang’s method, the self-relationships of individuals that carry more rare variants are inflated. Moreover, relatedness between two individuals that carry one or more copies of a rare variant will be higher than that of two individuals that carry a common variant. Consequently, selection decisions, for only a subset of individuals, based on relationships estimated with Yang’s method will increasingly favour individuals that share more common variants compared to when they are based on the similarity-based method. This property of Yang’s method reduces the potential for conservation of rare variants, making it suboptimal in the context of genetic diversity conservation.

### Optimal contribution selection

It has previously been shown that OC selection has a higher potential than random selection or traditional selection methods for genetic diversity conservation by yielding lower rates of inbreeding, a smaller loss of founder alleles [41] or a lower percentage of fixed alleles [9]. In our study, we were able to quantify the level of genetic diversity with a higher resolution by using WGS data. One striking conclusion was the important loss of genetic diversity at rare variants due to selection in both *cons* and *impcons* strategies. Stringent selection, such as selection of only five individuals in our analyses, is not advisable for prioritisation decisions in conservation or genetic improvement strategies since it causes a dramatic loss of genetic diversity and a steep increase in the rate of inbreeding.

As in Engelsma et al. [9], we observed that using genomic information for OC did, in general, conserve more genetic diversity than pedigree-based OC. In addition, we showed that, overall, OC using WGS data conserved slightly more genetic diversity than OC using SNP chip information, and that this difference was more specifically due to the conservation of more rare variants. With the *cons* strategy, using estimated relationships based on WGS data conserved more rare variants than when using relationships based on SNP chip data. With the *impcons* strategy, we found that using 50 K SNP chip data was sufficient to conserve a large number of common variants but that WGS data were more efficient to conserve rare variants. In conclusion, the potential of OC to increase conservation of genetic diversity is slightly higher with WGS data than with pedigree or SNP chip data.

### Measures of genetic diversity

In this study, our interest was directed to the conservation of rare variants since they have a greater chance to be lost either because of artificial or natural selection or random genetic drift [42]. Different methods can be used to measure genetic diversity, such as proportion of polymorphic loci, percentage of fixed alleles, expected and observed heterozygosity, rate of inbreeding, or number of alleles per locus (For an overview, see: [27]). As mentioned by Jobling et al. [43], the reliability of measures of genetic diversity based on genomic information depends on the density of the genomic information used. We measured the amount of genetic diversity conserved by the number of variants that continued to segregate after selection i.e. all variants (MAF ≥ 1 %), common variants (MAF ≥ 5 %) and rare variants (MAF between 1 and 5 %). This measure is equivalent to the proportion of polymorphic loci and opposite to the percentage of fixed alleles. The number of segregating variants has been used as a measure of genetic diversity before [44], and is a principal component of the Tajima’s D estimate of diversity [45]. As shown in our study, using WGS data to measure genetic diversity sheds light on the important loss of genetic diversity due to selection, especially at rare variants, that have the highest risk to be lost.

## Conclusions

This study showed that, depending on the number of individuals selected, dramatic losses of rare variants due to selection can be observed, with losses up to 72 % across the considered selection strategies based on optimal contribution (OC). Such losses of rare variants are not observed when using SNP chip data to measure genetic diversity, because the construction of SNP chips usually focuses on variants with common rather than rare alleles. In general, the overall level of genetic diversity was slightly higher when using estimated genomic relationships compared to pedigree relationships in OC. Among the methods considered to estimate genomic relationships, the similarity-based relationships resulted in the largest amount of genetic diversity conserved in both strategies that target genetic improvement and conservation, or conservation alone. In the *cons* strategy that targets conservation only, using estimated relationships based on WGS data to perform selection resulted in the largest number of variants still segregating after selection, especially for rare variants. In the *impcons* strategy that targets both genetic improvement and conservation, using estimated relationships based on SNP chip or WGS data resulted, respectively, in the largest number of common or rare variants still segregating after selection. Using WGS data slightly reduced the loss of rare variants, while 50 K SNP chip data was sufficient to conserve common variants. The large loss of genetic diversity due to loss of rare variants indicates that conservation decisions should put more emphasis on these variants. These findings should be considered in the development of breeding strategies in the context of genetic diversity conservation.

## Additional files

10.1186/s12711-016-0210-4 Comparison of **G**-matrices. Comparison of different methods to calculate estimated relationships between individuals and their impact on the loss of genetic diversity.
10.1186/s12711-016-0210-4 Pedigree subsets. Impact of using different pedigree subsets that are defined based on depth and completeness on genetic diversity conservation.
