# Supplementary material for: Whole-genome sequence data uncover loss of genetic diversity due to selection
Source: Genet Sel Evol. 2016 Apr 14;48:33. doi: 10.1186/s12711-016-0210-4 (PMC4831198; doi:10.1186/s12711-016-0210-4)
Supplement: Supplementary file 1 — 10.1186/s12711-016-0210-4 Comparison of G-matrices. Comparison of different methods to calculate estimated relationships between individuals and their impact on the loss of genetic diversity. [file 12711_2016_210_MOESM1_ESM.pdf]

Additional file 1 – G-matrices comparison

Introduction

Multiple methods are currently available and used to calculate estimated relationships. However there is nowadays no consensus on which of these methods is the most appropriate in the context of genetic diversity. Here we compare, on one simple case, different methods to calculate **G**-matrices in order to select the most appropriate for further analysis.

Methods

G-matrices were calculated in four different ways. Using the VanRaden methods [1, 2], using the Yang's method [3] derived for the second VanRaden method and finally using a method fixing allele frequencies to a unique value, 0.5, so similarity like method [4]. Whole Genome Sequence data was always used to estimate relationships.

Optimal Contribution (OC) selection was performed using the program Gencont [5] in the context of genetic diversity conservation. The optimum number, 20, 10 or five individuals were selected and the number of variants still segregating after selection was measure as a proxy for genetic diversity.

Results

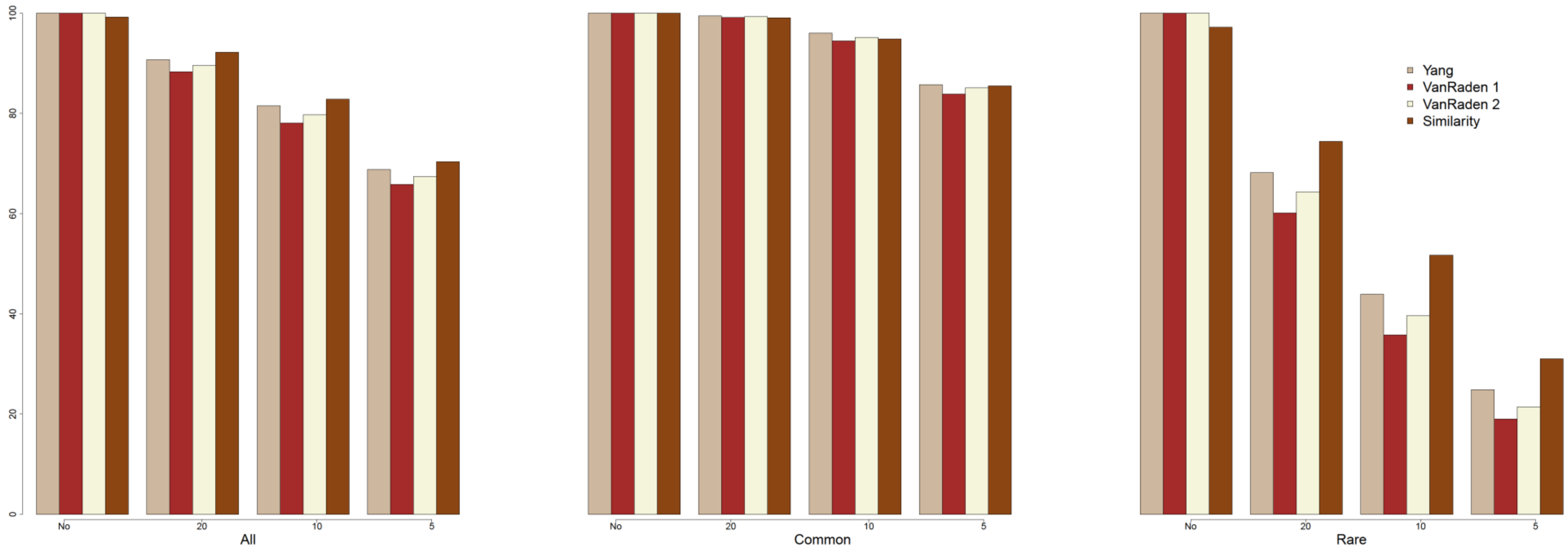

In most cases and especially at rare variants the Yang's method and the method based on similarity allowed conservation of more genetic diversity than both the VanRaden methods.

Conclusions

The VanRaden methods seem suboptimal for genetic diversity conservation compared to the Yang's method and the method based on similarity. After this analysis the two last methods were kept and compared in the rest of the study.

1. VanRaden PM. Efficient methods to compute genomic predictions. J Dairy Sci. 2008;91:4414-23.
2. VanRaden PM, Olson KM, Wiggans GR, Cole JB, Tooker ME. Genomic inbreeding and relationships among Holsteins, Jerseys, and Brown Swiss. J Dairy Sci. 2011;94:5673-82.
3. Yang JA, Benyamin B, McEvoy BP, Gordon S, Henders AK, Nyholt DR, et al. Common SNPs explain a large proportion of the heritability for human height. Nat Genet. 2010;42:565-9.
4. Eynard SE, Windig JJ, Leroy G, van Binsbergen R, Calus MPL. The effect of rare alleles on estimated genomic relationships from whole genome sequence data. BMC Genet. 2015;16:12.
5. Meuwissen THE. Maximizing the response of selection with a predefined rate of i nbreeding. J Anim Sci. 1997;75:934-40.
