# Supplementary material for: Whole-genome sequence data uncover loss of genetic diversity due to selection
Source: Genet Sel Evol. 2016 Apr 14;48:33. doi: 10.1186/s12711-016-0210-4 (PMC4831198; doi:10.1186/s12711-016-0210-4)
Supplement: Supplementary file 2 — 10.1186/s12711-016-0210-4 Pedigree subsets. Impact of using different pedigree subsets that are defined based on depth and completeness on genetic diversity conservation. [file 12711_2016_210_MOESM2_ESM.pdf]

Additional file 2 – Pedigree subsets

Introduction

Pedigree depth and completeness are important characteristics influencing estimated relationships between individuals. A deeper or more complete pedigree will give more accurate information on the relationships between individuals than a shallow pedigree [1].

Methods

We calculated estimated relationships, i.e. the A matrix, based on the complete pedigree and two pedigree subsets. In the complete pedigree for 268 individuals with EBVs, each individual had at least a full record on its parental generation. The subset Pedigree(1) was restricted to individuals that had at least a generation equivalent of six, and contained 263 bulls having EBV records. The subset Pedigree(2) was restricted to individuals having at least full records on both parents and grand-parents, as well as a generation equivalent of minimum six, this subset contained 125 individuals with EBV records.

Using these different pedigree subsets we performed OC selection in the different cases reported in our study. We measured the percentage of variants still segregating after selection relative to the number of variants segregating before selection in the complete pedigree. The results were evaluated on three different types of variants: rare variants (MAF between 1 % and 5 %), common variants (MAF ≥ 5 %) and all variants (MAF ≥ 1 %).

Results

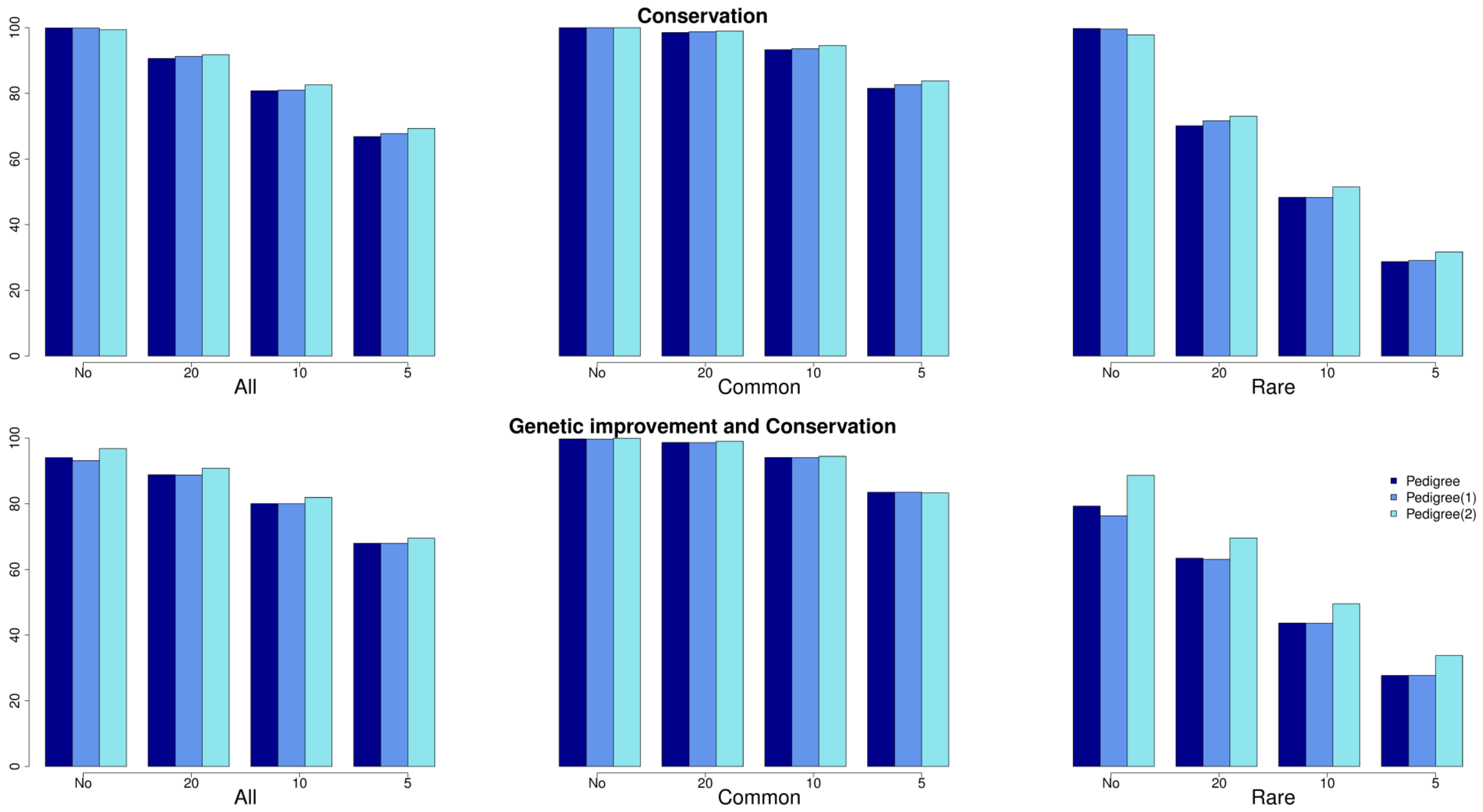

Conservation
Genetic improvement and Conservation
100
80
60
40
20
0
No
20
10
5
All
Common
Rare
Pedigree
Pedigree(1)
Pedigree(2)

Slight differences could be seen between the three pedigrees. Although Pedigree(2) was a subset of the complete pedigree and had less individuals to carry out selection on it performed better to conserve genetic diversity than the complete pedigree and Pedigree(1) when the restriction on number of selected individuals was more stringent, especially at rare variants. The differences between pedigrees were bigger for the genetic improvement and conservation strategy than for the conservation strategy.

Conclusions

A deeper and more complete pedigree allows a more accurate decision for genetic diversity conservation when the restriction on number of selected individuals is strict and for rare variants. When a large group or even all the individuals can potentially be kept after selection both pedigrees seem to perform equally well. For our analysis we only used the complete pedigree because it contains all the individuals and allowed direct comparison with the prioritisation decision based on genomic information without an extensive loss of genetic diversity conservation.

1. Sorensen MK, Sorensen AC, Baumung R, Borchersen S, Berg P. Optimal genetic contribution selection in Danish Holstein depends on pedigree quality. Livest Sci. 2008;118:212-22.
